# Ambient Air Pollution Shapes Bacterial and Fungal Ivy Leaf Communities

**DOI:** 10.3390/microorganisms9102088

**Published:** 2021-10-03

**Authors:** Vincent Stevens, Sofie Thijs, Eva Bongaerts, Tim Nawrot, Wouter Marchal, Jonathan Van Hamme, Jaco Vangronsveld

**Affiliations:** 1 Center for Environmental Sciences, Environmental Biology, Hasselt University, 3590 Diepenbeek, Belgium; sofie.thijs@uhasselt.be (S.T.); eva.bongaerts@uhasselt.be (E.B.); tim.nawrot@uhasselt.be (T.N.); jaco.vangronsveld@uhasselt.be (J.V.); 2 Institute for Materials Research, Analytical and Circular Chemistry, Hasselt University, 3590 Diepenbeek, Belgium; wouter.marchal@uhasselt.be; 3 TRUGen Applied Genomics Laboratory, Department of Biological Sciences, Thompson Rivers University, Kamloops, BC V2C 0C8, Canada; jvanhamme@tru.ca; 4 Department of Plant Physiology and Biophysics, Faculty of Biology and Biotechnology, Maria Curie–Skłodowska University, 20-400 Lublin, Poland

**Keywords:** ambient air pollution, black carbon, *Hedera helix*, phylloplane, microbial communities, metabarcoding, BTX degradation

## Abstract

Ambient air pollution exerts deleterious effects on our environment. Continuously exposed to the atmosphere, diverse communities of microorganisms thrive on leaf surfaces, the phylloplane. The composition of these communities is dynamic, responding to many environmental factors including ambient air pollution. In this field study, over a 2 year period, we sampled *Hedera helix* (ivy) leaves at six locations exposed to different ambient air pollution conditions. Daily, we monitored ambient black carbon (BC), PM_2.5_, PM_10_, nitrogen dioxide, and ozone concentrations and found that ambient air pollution led to a 2–7-fold BC increase on leaves, the phylloplane BC load. Our results further indicated that the phylloplane BC load correlates with the diversity of bacterial and fungal leaf communities, impacting diversity more than seasonal effects. The bacterial genera *Novosphingobium*, *Hymenobacter*, and *Methylorubrum*, and the fungal genus *Ampelomyces* were indicators for communities exposed to the highest phylloplane BC load. Parallel to this, we present one fungal and two bacterial phylloplane strains isolated from an air-polluted environment able to degrade benzene, toluene, and/or xylene, including a genomics-based description of the degradation pathways involved. The findings of this study suggest that ambient air pollution shapes microbial leaf communities, by affecting diversity and supporting members able to degrade airborne pollutants.

## 1. Introduction

Ninety-one percent of the world’s human population is currently living in places where air pollution parameters exceed World Health Organization (WHO) guideline limits [1]. It is most pronounced in high-traffic environments such as urban areas where substantial quantities of airborne pollutants, including particulate matter (PM) and volatile organic compounds (VOCs), are generated [2]. Black carbon (BC) is a ubiquitous component of ambient PM that is produced during incomplete combustion associated with biomass burning, transportation, residential activities, and industry [3], and it has a number of adverse public health and environmental impacts [4]. For example, considerable damage is caused to plants as a result of PM/BC deposition, leading to inhibition of photosynthetic activities and protein synthesis, as well as increased susceptibility to injuries caused by microorganisms and insects [5]. VOCs can be defined as organic compounds having a vapor pressure of more than or equal to 0.01 kPa at 20 °C, or having a corresponding volatility; this includes most polycyclic aromatic hydrocarbons containing up to four aromatic rings. Important fossil fuel-related VOCs include benzene, toluene, and xylene (BTX), 1,3-butadiene, ethylbenzene, styrene, naphthalene, and phenanthrene [6,7]. In addition to their role in ozone formation, these VOCs are reported to induce both short- and long-term adverse health effects including asthma, allergies, and cancer [8,9].

Always in contact with the surrounding air, abundant and diverse communities of microorganisms naturally exist on the surface of above-ground parts of plants, known as the phyllosphere [10]. The phyllosphere can be subdivided into the caulosphere (stems), phylloplane (leaves), anthosphere (flowers), and carposphere (fruits). With an estimated global leaf surface area of 1,017,260,200 km^2^ [11], the phylloplane is one of the most prevalent microbial habitats on earth, with bacteria being by far the most abundant and persistent organisms, at typical densities of 10^6^–10^7^ cells·cm^−2^ [12]. Most studies on the identity of organisms in the phyllosphere have focused on bacteria and, to a lesser extent, fungi, which are typically less numerous [10]. The composition of these microbial communities is dynamic, responding to environmental factors, both biotic and abiotic, such as leaf age, the copresence of other (micro)organisms, ultraviolet (UV) light exposure, fertilization, and water limitation [13].

It is evident that ambient air pollution not only poses hazards to humans, animals, and plants, but also influences phylloplane microbial communities, as leaf surfaces are continuously exposed to the atmosphere. However, to date, there has been little research done on the extent and nature of this influence. Here, we deployed metabarcoding to study the effects of ambient air pollution in the field on bacterial and fungal communities in the phylloplane of *Hedera helix* (ivy), an evergreen plant known for its hardiness and climbing ability [14]. *H. helix* has a widespread distribution in the northern hemisphere in diverse environments such as private gardens, city centers, municipal parks, nature reserves, and forests, and it shows a high capacity to take up and filter pollutants out of the air [15]. Over eight sampling events during a 2 year period, we sampled 4320 *H. helix* leaves at six locations exposed to different ambient air pollution conditions. We characterized these locations daily by monitoring ambient BC, PM_2.5_, PM_10_, nitrogen dioxide, and ozone concentrations. Furthermore, we measured phylloplane BC load and carried out extensive biennial metabarcoding surveys. In addition, we here present three phylloplane microorganisms isolated from an air-polluted environment that are able to degrade benzene, toluene, and xylene, including a genomics-based description of the degradation pathways involved.

## 2. Materials and Methods

### 2.1. Sampling Site Characteristics

We selected six sampling sites around Hasselt, Belgium harboring stable and healthy populations of *H. helix* plants. WGS84 coordinates were as follows: 50.936546, 5.317226 (HGa); 50.917344, 5.326918 (HGb); 50.934242, 5.337944 (HKa); 50.928680, 5.332674 (HKb); 50.940104, 5.438675 (DM); 50.921694, 5.433951 (DW). HGa and HGb are located on the outer city ring road of Hasselt, HKa and HKb are located on the inner city ring road, and DM and DW are located in two close-by nature reserves (Figure S1). The soil type at the six sites was characterized to be sandy loam with an average pH of 6.46 ± 0.1 (HGa: 6.41 ± 0.02, HGb: 6.38 ± 0.03, HKa: 6.61 ± 0.04, HKb: 6.56 ± 0.02, DM: 6.42 ± 0.01, DW: 6.37 ± 0.02) and average soil organic matter content of 962 ± 131 mg·kg^−1^ (HGa: 766 ± 61 mg·kg^−1^, HGb: 1022 ± 45 mg·kg^−1^, HKa: 914 ± 64 mg·kg^−1^, HKb: 932 ± 28 mg·kg^−1^, DM: 1169 ± 35 mg·kg^−1^, DW: 965 ± 26 mg·kg^−1^). Permission for sampling was obtained, and it was performed in accordance with institutional and international guidelines. Black carbon (BC), PM_2.5_, PM_10_, nitrogen dioxide, and ozone concentrations were monitored daily by the Belgian Interregional Environment Agency from 1 August 2017 until 31 August 2019 (*n* = 761) and calculated for every sampling site using the RIO–IFDM model [16]. Briefly, air pollution data of the official Belgian fixed monitoring network are interpolated using land-cover data from satellite images from the CORINE dataset [17], in combination with a dispersion model [18]. This model provides interpolated air pollution values from the Belgian telemetric air quality networks, “point” sources such as industries, and “line” sources such as highways, on a dense, irregular receptor point grid with a max cell size of 25 × 25 m. The performance of the overall model was assessed by a leave-one-out cross-validation; the validation statistics explained more than 74% of the spatiotemporal variability for BC, 80% for PM_2.5_ and PM_10_, 78% for nitrogen dioxide, and 65% for ozone [19]. Differences in mean air pollution parameter values between sampling sites were analyzed by performing analysis of variance (ANOVA) and multiple *t*-tests with Bonferroni correction. Temperature and precipitation were also monitored daily for this period by an automated weather station owned by the Royal Meteorological Institute of Belgium (WGS84 coordinates: 50.911774, 5.406425).

### 2.2. Collection and Preparation of Phylloplane Samples

Leaves (*n* = 3840; 80 per site, per sampling round) from *H*. *helix* plants ranging in age from 3–6 months were collected during eight sampling events, one per season, over the course of two seasonal cycles (years): 14 November 2017 (I); 5 February 2018 (II); 14 May 2018 (III); 1 August 2018 (IV); 5 November 2018 (V); 19 February 2019 (VI); 21 May 2019 (VII); 23 August 2019 (VIII). Plant leaves were identified as specimens belonging to *H*. *helix* by the first author; voucher specimens are available from Hasselt University. Leaves were cut from the plants at shoulder height using sterile forceps, put into sterile tubes (five leaves per tube) filled with phosphate buffer (50 mM Na_2_HPO_4_∙7H_2_O, 50 mM NaH_2_PO_4_∙H_2_O, 0.8 mM Tween-80, pH 7.0), and immediately transferred to the laboratory. Leaf weight was determined gravimetrically, and microbial cells were detached from the leaf surface by sonication (100 W, 42 kHz, 3 min), followed by shaking on an orbital shaker (240 rpm, 30 min). Next, resulting leaf wash suspensions (16 per site, per sampling round) were centrifuged (3000× *g*, 15 min), and the resuspended pellets were pooled into four samples per site, per sampling round, resulting in a total of 192 samples. Samples were immediately stored at −80 °C until DNA isolation.

An additional set of *H. helix* leaves (*n* = 480; 10 per site, per sampling round) was collected as previously described, except the resuspended pellets were not pooled, resulting in a total of 96 samples (two per site, per sampling round). Leaf wash suspensions were immediately stored at −20 °C until BC detection.

### 2.3. Black Carbon Detection in Leaf Wash Suspensions

BC particles present in leaf wash suspensions (*n* = 96) were detected using a specific and sensitive detection technique based on the non-incandescence-related white light (WL) generation of the particles under femtosecond-pulsed illumination, as previously described [20]. The day before measuring, leaf wash suspensions were transferred from −20 °C to room temperature. Z-stacks of the samples were collected using a Zeiss LSM 880 (Carl Zeiss, Oberkochen, Germany) equipped with a femtosecond-pulsed laser (810 nm, 150 fs, 80 MHz, Mai Tai DeepSee, Spectra–Physics, Andover, MA, USA) tuned to a central wavelength of 810 nm using a plan-apochromat 20× (0.8 NA) objective (Carl Zeiss, Oberkochen, Germany). Two-photon-induced WL emission of BC particles was acquired in the non-descanned mode after spectral separation and emission filtering using 400–410 nm and 450–650 nm bandpass filters. Each sample was aliquoted at 20 µL on a glass coverslip, and z-stacks were collected from the bottom up to 35 µm in the droplet. The resulting z-stacks had an imaging volume of 425.1 × 425.1 × 35.1 μm^3^ and a pixel dwell time of 1.54 µs. In total, 175 images per perfusate sample were obtained by recording z-stacks with a 1 µm slice thickness at five different locations in the aliquot (*n* = 480). The images were acquired using ZEN Black 2.0 software (Carl Zeiss, Oberkochen, Germany).

The number of BC particles in the obtained z-stacks was determined using a custom Matlab program (Matlab 2010, MathWorks, Eindhoven, The Netherlands). First, a peak-searching algorithm counted pixels above a threshold value. Here, threshold values of 0.1% lower than the highest pixel intensity value of the narrow second-harmonic generation channel (405/10) and two-photon-excited autofluorescence channel (550/200) were chosen. Next, the thresholded pixels of both images were compared, and only the overlapping ones were counted as BC particles. The average number of particles detected in the washing sample z-stacks was normalized to the imaging volume, and the results were expressed as the number of detected BC particles per g leaf material. Differences in mean values between sampling sites were analyzed by performing ANOVA and multiple *t*-tests with Benjamini–Hochberg correction.

### 2.4. Metabarcoding of the Bacterial and Fungal Phylloplane

Leaf wash suspensions (*n* = 192) were centrifuged (10,000× *g*, 10 min, 4 °C), and genomic DNA was isolated using a NucleoSpin Soil kit (Macherey–Nagel, Düren, Germany). Initially, two different sequencing libraries were prepared, one for bacterial and one for fungal phylloplane metabarcoding. The bacterial sequencing library was constructed by PCR amplification of the V3–V4 hypervariable region of the 16S rRNA gene using 341F (5′–TCGTCGGCAGCGTCAGATGTGTATAAGAGACAGCCTACGGGNGGCWGCAG–3′) and 785R (5′–GTCTCGTGGGCTCGGAGATGTGTATAAGAGACAGGACTACHVGGGTATCTAATCC–3′) primers [21] containing Nextera (Illumina, San Diego, CA, USA) transposase adapters (underlined). For fungal sequencing library construction, the hypervariable internal transcribed spacer 2 (ITS2) region between the 5.8S and 28S rRNA genes was PCR-amplified using gITS86F (5′–TCGTCGGCAGCGTCAGATGTGTATAAGAGACAGGTGARTCATCGARTCTTTGAA–3′) and ITS4R (5′–GTCTCGTGGGCTCGGAGATGTGTATAAGAGACAGTCCTCCGCTTATTGATATGC–3′) primers [22] including the same adapters. The 25 µL PCR reaction consisted of 1× reaction buffer with 1.8 mM MgCl_2_, 0.2 µM dNTP mix, 0.05 U·µL^−1^ enzyme blend (FastStart High Fidelity PCR System, Roche, Basel, Switzerland), 0.2 µM of each primer, 5% DMSO, and 1 µL of DNA template. Amplification conditions were as follows: 95 °C for 2 min; 25 cycles of 95 °C for 30 s, 54/58 °C (bacterial/fungal sequencing library) for 30 s, 72 °C for 1 min; held at 72 °C for 6 min. PCR products were purified using AMPure XP beads (Beckman Coulter, Brea, CA, USA) and subsequently indexed (*n* = 384) using the Nextera XT index kit (Illumina, San Diego, CA, USA). The 25 µL index PCR reaction consisted of 1× reaction buffer with 1.8 mM MgCl_2_, 0.2 µM dNTP mix, 0.05 U·µL^−1^ enzyme blend (FastStart High Fidelity PCR System, Roche, Basel, Switzerland), 0.2 µM of each index primer, and 1 µL of purified PCR product. Amplification conditions were as follows: 95 °C for 2 min; 14 cycles of 95 °C for 30 s, 55 °C for 30 s, 72 °C for 1 min; held at 72 °C for 6 min. After another round of purification, the DNA concentration of every indexed sample was quantified with a Qubit dsDNA HS assay kit and the Qubit 2.0 fluorometer (Thermo Fisher Scientific, Waltham, MA, USA) prior to equimolar pooling of the samples. Correct amplicon size and integrity were checked on an Agilent 2100 Bioanalyzer system (Agilent Technologies, Santa Clara, CA, USA), followed by sequencing using a MiSeq Reagent Kit v3 on a MiSeq system (Illumina, San Diego, CA, USA) at Hasselt University, Belgium. When preparing the sequencing libraries, a mock community containing eight bacterial and two fungal strains was included to assess sequencing biases, errors, and other artefacts (ZymoBIOMICS Microbial Community DNA Standard, Zymo Research, Irvine, CA, USA).

### 2.5. Analysis of Sequencing Data

Demultiplexed metabarcoding data were received in FASTQ format with all additional nucleotides removed except the 341F/785R (bacteria) or gITS86F/ITS4R (fungi) primers, and they were further processed with DADA2 1.18.0 [23]. Primer sequences were removed using cutadapt 2.1 [24], ShortRead 1.50.0 [25], and Biostrings 2.60.1 (R package). Forward reads were quality-filtered by truncation to 260/230 bp (bacteria/fungi) and reverse reads were quality-filtered by truncation to 190/160 bp, with maxN = 0 (max number of Ns) and maxEE = 1 (max number of expected errors). Default DADA2 parameter settings were used for error model learning, dereplication, amplicon sequence variant (ASV) inference, merging of forward and reverse reads, and chimera removal. Taxonomy was assigned to each resulting ASV with IDTAXA [26] using the RDP v18 16S rRNA [27] or Warcup v2 ITS [28] training set. ASV tables with assigned taxonomic entities were imported into phyloseq 1.36.0 [29], chloroplast sequences were discarded, and samples with a read count of less than 500 were removed, resulting in a dataset of 190/192 (bacteria/fungi) samples. Intra-sample (alpha) diversity was assessed using ASV observations and by calculating Shannon diversity indices, and rarefaction analyses were performed with ranacapa 0.1.0 [30]. Differences in alpha diversity mean values were analyzed by performing ANOVA and multiple *t*-tests with Benjamini–Hochberg correction. Before inter-sample (beta) diversity was measured, counts in every sample were scaled by $S\left(x\right)=\log_{10}\left(x+1\right)$ and normalized to even sampling depth by $N\left(x\right)=\frac{x}{\sum_{i=1}^{n}x}\;10^{6}$, where *n* = 190/192 (bacteria/fungi). Principal coordinate analysis (PCoA) and nonmetric multidimensional scaling (NMDS) and was performed on Bray–Curtis dissimilarity matrices, and different outcomes were tested by geometric partitioning of variation with analysis of similarities (ANOSIM) and permutational multivariate analysis of variance (PERMANOVA), using vegan 2.5–7 (R package). Differentially abundant taxa between designated categories were identified by linear discriminative analysis (LDA) with effect size [31], where the Kruskal–Wallis test was used to detect taxa with significant (*p* < 0.05) differential abundance between the indicated categories. Next, LDA was used to estimate the effect size of each differentially abundant taxon; only LDA log_10_ scores ≥2 were considered. All mentioned data handling, statistics, and creation of plots (ggplot2 3.3.5, R package) were done within R 4.1.0 [32].

### 2.6. Benzene, Toluene, and Xylene Degradation by Phylloplane Isolates

First, we screened a collection (*n* = 300) of *H. helix* phylloplane isolates for their potential to metabolize diesel fuel (DF). Individual cultures were grown for 24 h in LB medium [33] at 30 °C on a shaker (150 rpm) until a culture density (OD_600 nm_) was reached between 0.8 and 1.2, pelleted by centrifugation, washed three times, and resuspended in 0.01 M phosphate-buffered saline (PBS). Culture suspensions were left overnight at 30 °C on a shaker (150 rpm). The next day, 80 µL of the suspensions were transferred to 750 µL of Bushnell–Haas (BH) medium [34] with the addition of 50 µL of filter-sterilized 100 µg·mL^−1^ 2,6-dichlorophenolindophenol (DCPIP) and 5 µL of polytetrafluoroethylene (PTFE) filter-sterilized 0.45 µM DF as the sole carbon source [35]. After incubation in the dark for 5 days at 30 °C on a shaker (150 rpm), suspensions showing a visible change from blue to colorless (due to reduction of DCPIP, indicating respiration) were evaluated as positive for DF metabolization. The first part (*n* = 200) of this collection containing isolates sampled from HGa, HKb, DM, and DW was previously described [36] and resulted in two DF metabolizers [37,38], both isolated from HKb. The second part (*n* = 100), comprising isolates sampled only from HKb (isolated in the same way as described before [36], but using only LB medium), was screened and resulted in another four DF metabolizers. For long-term storage, isolates were flash-frozen in a solution of 30% (*v*/*v*) glycerol and 0.15 M NaCl, and stored at −45 °C.

Our screening effort resulted in six DF metabolizers which were further analyzed for their potential to degrade benzene, toluene, and xylene (BTX) using headspace gas chromatography–mass spectrometry (GC–MS). Individual cultures were grown for 24 h in LB medium at 30 °C on a shaker (150 rpm) until a culture density (OD_600 nm_) was reached between 0.8 and 1.2, pelleted by centrifugation, washed three times, and resuspended with BH medium. Using 20 mL headspace GC–MS vials, benzene, toluene, or *ortho*-, *meta*-, *para*-xylene was added to one series of BH medium and one series of BH medium with additional macronutrients (0.5 g·L^−1^ yeast extract, 0.5 g·L^−1^ tryptone, and 0.5 g·L^−1^ d-glucose) resulting in a final concentration of 20 mg·L^−1^ benzene, toluene, or xylene in 8 mL of medium including 800 µL of bacterial suspension. Vials were incubated in the dark for 10 days at 30 °C on a shaker (100 rpm). With regard to GC–MS analysis, samples were incubated at 80 °C for 20 min, and 1 mL of the headspace was injected into a TRACE 1310 gas chromatograph coupled to an ISQ single quadrupole GC–MS system (Thermo Fisher Scientific, Waltham, MA, USA).

### 2.7. Genome Sequencing and Analysis

We sequenced the genomes of two bacteria and one yeast that were able to degrade benzene, toluene, and/or xylene. Individual cultures were grown for 24 h in LB medium at 30 °C on a shaker (150 rpm) and centrifuged (10,000× *g*, 3 min); then, genomic DNA was isolated from the pellets using the DNeasy Blood & Tissue kit (QIAGEN, Hilden, Germany). The DNA concentration was quantified with a Qubit dsDNA HS assay kit and the Qubit 2.0 fluorometer (Thermo Fisher Scientific, Waltham, MA, USA). The Nextera DNA Flex library preparation kit along with the Nextera DNA CD indices (Illumina, San Diego, CA, USA) were used to tagment the DNA libraries for sequencing following the manufacturer’s instructions. Tagmentation was performed with 100 ng of genomic DNA, and amplification conditions were as follows: 68 °C for 3 min; 98 °C for 3 min; five cycles of 98 °C for 45 s, 62 °C for 30 s, 68 °C for 2 min; 68 °C for 1 min. The DNA concentration of every indexed sample was quantified as previously described prior to equimolar pooling of the samples. Library size distribution was checked on an Agilent 2100 Bioanalyzer system (Agilent Technologies, Santa Clara, CA, USA), followed by sequencing on a HiSeq X Ten system (Illumina, San Diego, CA, USA) by Macrogen (Seoul, South Korea).

Paired-end reads were assembled de novo using SPAdes 3.15.2 [39]. Contigs with a length of less than 1000 bp were discarded and genome assemblies were evaluated with QUAST 5.0.2 [40]. Bacterial genome annotation was completed with Prokka 1.14.6 [41] using the TIGRFAMs [42], Pfam [43], and HAMAP [44] hidden Markov model (HMM)-based databases. AUGUSTUS 3.4.0 [45] was used for fungal genome annotation. KEGG [46] orthology (KO) identifiers were assigned to individual genes with BlastKOALA 2.2 [47], followed by reconstruction of KEGG pathways using KEGG Mapper [48].

## 3. Results

### 3.1. Ambient Air Pollution Increases Black Carbon Load in the Phylloplane

**Figure 1:**
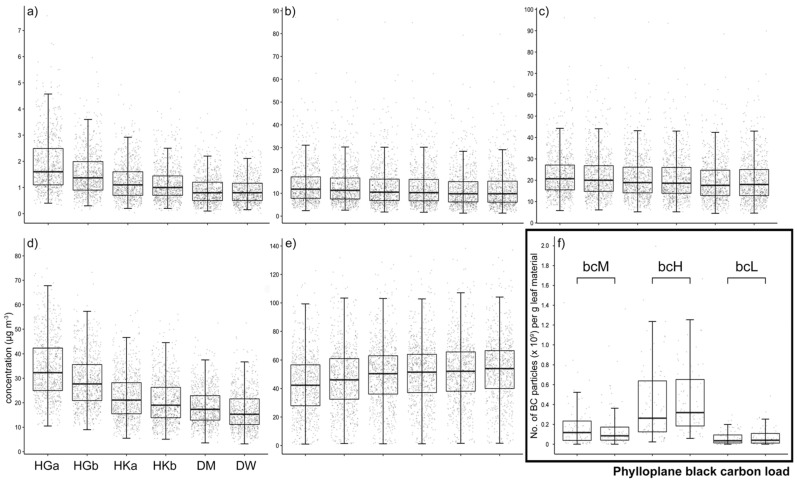
Ambient air pollution parameters and phylloplane black carbon load. Ambient BC (**a**), PM_2.5_ (**b**), PM_10_ (**c**), nitrogen dioxide (**d**), and ozone (**e**) concentrations were monitored daily from 1 August 2017 until 31 August 2019 (*n* = 761) at six sampling sites (HGa, HGb, HKa, HKb, DM, and DW) harboring populations of *H. helix* plants. Phylloplane BC load (**f**) was assessed during each sampling round (*n* = 480). Box plots span the interquartile range (25th to 75th percentile), lines within boxes denote the median, and whiskers extend to 1.5 times the interquartile range. Low (bcL), medium (bcM), and high (bcH) phylloplane BC load.

We monitored ambient BC, PM_2.5_, PM_10_, nitrogen dioxide, and ozone concentrations on a daily basis over 2 years (from 1 August 2017 until 31 August 2019) at six sampling sites (Figure 1a–e). We also monitored temperature and precipitation during this period (Figure S2). A cyclical (seasonal) pattern was observed during these 2 years, with BC peaking in winter, PM_2.5_ and PM_10_ peaking during late winter/early spring, and NO_2_ and O_3_ peaking in winter and summer, respectively (Figure S3). Overall, significant (*p* < 0.05) differences were observed for the six sampling sites for a number of parameters (Table 1). For example, BC, PM_2.5_, PM_10_, and NO_2_ concentrations were highest (and O_3_ concentration lowest) at HGa, followed by HGb and HKa,b. Conversely, the lowest concentrations for these parameters were detected at DM and DW, sites where O_3_ concentrations were highest. We also measured phylloplane BC load during each of the eight sampling events (Figure 1f, Table 1). Here, we observed the highest BC load for HKa,b (bcH). DM and DW had the lowest BC load (bcL), and leaves at HGa,b experienced an intermediate BC load (bcM). While our data show that higher concentrations of ambient BC and NO_2_ (and lower O_3_ concentrations) and, to a lesser extent, PM_2.5_ and PM_10_ correlate with a 2–7-fold increase in phylloplane BC load, other characteristics of the sampling sites appeared to have an influence. Notably, ambient air pollution was highest at HGa,b, while HKa,b had the highest phylloplane BC load.

### 3.2. Phylloplane Black Carbon Load Correlates with Bacterial and Fungal Diversity

**Figure 2:**
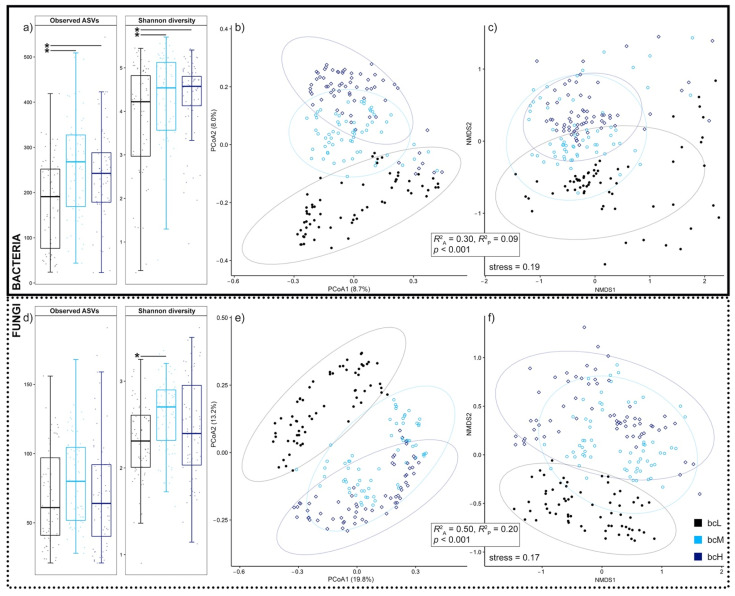
Phylloplane black carbon load correlates with bacterial and fungal diversity. Bacterial (*n* = 190) and fungal (*n* = 192) intra-sample (alpha) diversity was assessed with amplicon sequence variant (ASV) observations and by calculating Shannon diversity indices (**a**,**d**). Box plots span the interquartile range (25th to 75th percentile), lines within boxes denote the median, and whiskers extend to 1.5 times the interquartile range. Multiple *t*-test significance (*p* < 0.05) is indicated with an asterisk (*). Inter-sample (beta) diversity was measured with principal coordinate analysis (PCoA; **b**,**e**) and nonmetric multidimensional scaling (NMDS; **c**,**f**) on Bray–Curtis dissimilarity matrices. Ellipses indicate 95% data intervals. ANOSIM (*R*^2^_A_) and PERMANOVA (*R*^2^_P_) effect size and significance are shown. Low (bcL), medium (bcM), and high (bcH) phylloplane BC load.

Our metabarcoding effort of the bacterial and fungal phylloplane of *H. helix* plants, growing at six locations with different phylloplane BC loads due to differences in ambient air pollution, resulted in a dataset of 190/192 (bacteria/fungi) samples including 1,888,912 16S rRNA V3–V4/5,167,679 ITS2 sequences covering 15,610/1685 amplicon sequence variants (ASVs). Rarefaction analysis indicated all samples were sufficiently sequenced to represent their true biological identity (Figure S4). To test if phylloplane BC load influences the bacterial and fungal leaf microbiome of *H. helix*, we first estimated the intra-sample (alpha) diversity of bacteria and fungi by calculating two metrics: the observed number of ASVs and the Shannon diversity index. Bacterial alpha diversity was lower in samples with low phylloplane BC load (bcL), compared to samples with medium and high BC load (bcM and bcH, respectively) (Figure 2a). On the other hand, fungal alpha diversity was similar under all three phylloplane BC load conditions; only the Shannon diversity of bcL samples was lower compared to bcM samples (Figure 2d). Next, we inferred inter-sample (beta) diversity by employing PCoA and NMDS on Bray–Curtis dissimilarity matrices. These two-dimensional representations show significantly (*p* < 0.001) distinctive groupings depending on phylloplane BC load for both bacterial (Figure 2b,c) and fungal (Figure 2e,f) *H. helix* leaf communities (*R*^2^_ANOSIM_ = 0.30, *R*^2^_PERMANOVA_ = 0.09, bacteria; *R*^2^_ANOSIM_ = 0.50, *R*^2^_PERMANOVA_ = 0.20, fungi). Moreover, the calculated effect sizes show that the BC load-correlating shift on fungal diversity was greater than on bacterial diversity. Such distinctive groupings were absent for seasonal categorizations (Figure S5). However, the seasonal effects on bacterial and fungal beta diversity were significant (*p* < 0.001), albeit with much lower effect sizes than those of phylloplane BC load (*R*^2^_ANOSIM_ = 0.14, *R*^2^_PERMANOVA_ = 0.05, bacteria; *R*^2^_ANOSIM_ = 0.19, *R*^2^_PERMANOVA_ = 0.10, fungi). Our results show that phylloplane BC load, which was increased by ambient air pollution by 2–7-fold, strongly correlated with the diversity of bacterial and fungal *H. helix* leaf communities, and had more impact than seasonal effects, over a 2 year investigation period.

**Figure 3:**
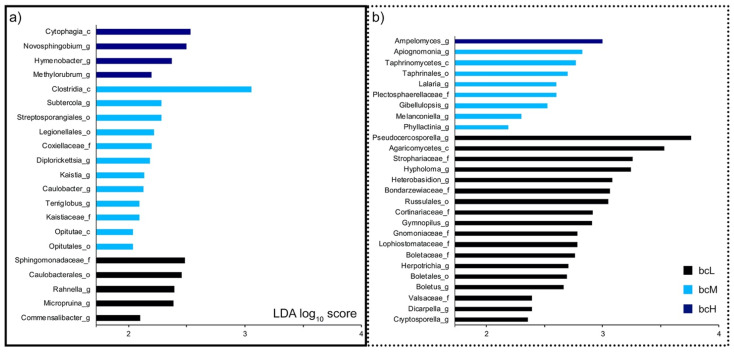
Indicators of bacterial and fungal leaf communities with different phylloplane black carbon load. Significant (*p* < 0.05, LDA with effect size) differentially abundant bacterial (**a**) and fungal (**b**) taxa between communities with low (bcL), medium (bcM), and high (bcH) phylloplane BC load. Only LDA log_10_ scores ≥2 were considered; a higher score denotes a more differentially abundant taxon. After every taxon name, the rank is indicated (c: class, o: order, f: family, g: genus).

To better understand the nature of these observed diversity shifts, we identified indicators by analyzing for significantly (*p* < 0.05) differentially abundant taxa (Figure 3). At the genus level, *Novosphingobium*, *Hymenobacter*, *Methylorubrum*, and *Ampelomyces* were indicators for communities with high phylloplane BC load (bcH). *Subtercola*, *Diplorickettsia*, *Kaistia*, *Caulobacter*, *Terriglobus*, *Apiognomonia*, *Lalaria*, *Gibellulopsis*, *Melanconiella*, and *Phyllactinia* were associated with communities with medium phylloplane BC load (bcM). *Rahnella*, *Micropruina*, *Commensalibacter*, *Pseudocercosporella*, *Hypholoma*, *Heterobasidion*, *Gymnopilus*, *Herpotrichia*, *Boletus*, *Dicarpella*, and *Cryptosporella* were indicators for communities with low phylloplane BC load (bcL). Indicators at higher taxonomic levels (family, order, class) were also identified. Class Cytophagia was an indicator for bcH phylloplane communities. Families Coxiellaceae, Kaistiaceae, and Plectosphaerellaceae, orders Streptosporangiales, Legionellales, Opitutales, and Taphrinales, and classes Clostridia, Opitutae, and Taphrinomycetes were associated with bcM phylloplane communities. Lastly, families Sphingomonadaceae, Strophariaceae, Bondarzewiaceae, Cortinariaceae, Gnomoniaceae, Lophiostomataceae, Boletaceae, and Valsaceae, orders Caulobacterales, Russulales, and Boletales, and class Agaricomycetes were indicators for bcL phylloplane communities.

### 3.3. Phylloplane Isolates from an Air-Polluted Environment Degrade BTX

**Figure 4:**
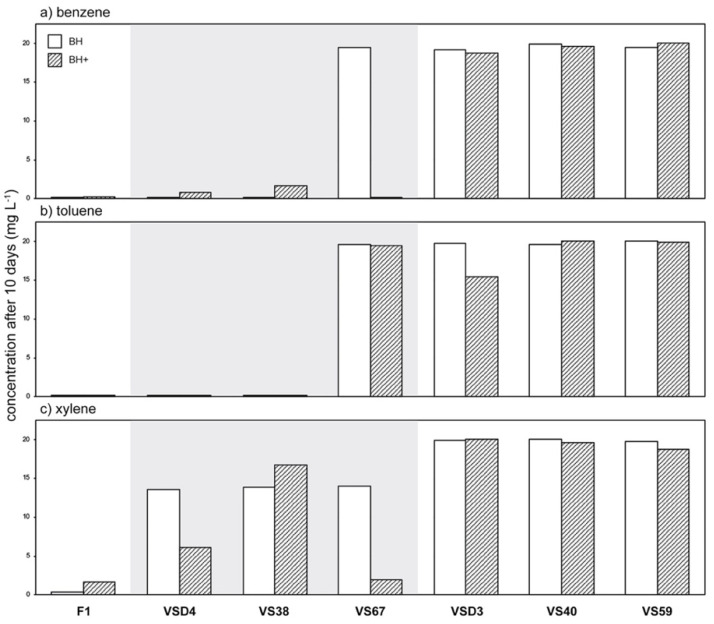
BTX degradation potential of phylloplane diesel fuel metabolizers. Six diesel fuel metabolizers isolated from the phylloplane of *H. helix* growing at an air-polluted environment were further analyzed by headspace GC–MS for their potential to degrade benzene (**a**), toluene (**b**), and xylene (**c**) (BTX): *Pseudomonas putida* F1 (positive control), *Bacillus licheniformis* VSD4, *Pseudomonas* sp. VS38, *Rhodotorula* sp. VS67, *Rhodococcus erythropolis* VSD3, *Pseudomonas* sp. VS40, and *Pseudomonas* sp. VS59. BH(+): Bushnell–Haas medium (+: with additional macronutrients; 0.5 g·L^−1^ yeast extract, 0.5 g·L^−1^ tryptone, and 0.5 g·L^−1^ d-glucose).

**Figure 5:**
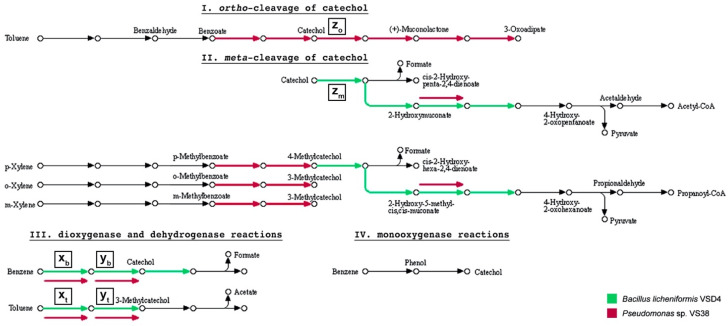
BTX degradation pathways. KEGG orthology (KO) identifiers were assigned to individual genes in the genomes of benzene, toluene, and/or *ortho(o)*-, *meta(m)*-, *para(p)*-xylene (BTX) degraders *Bacillus licheniformis* VSD4, *Pseudomonas* sp. VS38, and *Rhodotorula* sp. VS67, followed by reconstruction of KEGG pathways related to BTX degradation, categorized in the *ortho*- and *meta*-cleavage of catechol (I/II), dioxygenase, dehydrogenase (III), and monooxygenase (IV) reactions. Arrows highlighted in color indicate the presence of the respective enzyme-coding gene (homolog) in the specified genome. No enzyme-coding homologs related to these KEGG BTX degradation pathways were found for *Rhodotorula* sp. VS67. x_b/t_ benzene/toluene dioxygenase, y_b/t_ *cis*-benzene/toluene dihydrodiol dehydrogenase, z_o_ catechol 1,2-dioxygenase, z_m_ catechol 2,3-dioxygenase.

Given the impact of ambient air pollution on bacterial and fungal ivy leaf communities, it is reasonable to suggest that phylloplane microorganisms may be able to degrade common airborne pollutants. Therefore, we screened a collection of 300 *H. helix* phylloplane isolates for their potential to metabolize diesel fuel (DF). Our screening effort resulted in six DF metabolizers that were all isolated from bcH samples (HKb). We further analyzed these isolates for their potential to degrade benzene, toluene, and xylene (BTX). Two bacteria, *Bacillus licheniformis* VSD4 and *Pseudomonas* sp. VS38, were each able to completely degrade 20 mg·L^−1^ benzene and toluene in 10 days (Figure 4a,b). However, when additional carbon sources were provided, benzene removal was incomplete. Partial degradation of xylene was also observed for both isolates (Figure 4c). *Rhodotorula* sp. VS67, a red yeast, partially degraded xylene when it was provided as the sole carbon source. When other carbon sources where present, it was able to completely degrade benzene and (almost completely) xylene. Three of the diesel fuel metabolizers, *Rhodococcus erythropolis* VSD3, *Pseudomonas* sp. VS40, and *Pseudomonas* sp. VS59, were not able to notably degrade BTX. To better understand which degradation pathways are involved in the observed BTX degradation, we sequenced the genomes of *B. licheniformis* VSD4, *Pseudomonas* sp. VS38, and *Rhodotorula* sp. VS67 (Table S1). We assigned KEGG orthology (KO) identifiers to individual genes and reconstructed KEGG pathways related to BTX metabolism, categorized in the *ortho*- and *meta*-cleavage of catechol, dioxygenase, dehydrogenase, and monooxygenase reactions (Figure 5). The presence of homologs encoding the alpha subunit of benzene/toluene dioxygenase and *cis*-benzene/toluene dihydrodiol dehydrogenase was confirmed in silico in the genomes of *B. licheniformis* VSD4 and *Pseudomonas* sp. VS38. These two enzymes catalyze the oxygenation and dehydrogenation of benzene and toluene to catechol and 3-methylcatechol, respectively. From here on, degradation pathways in the two organisms diverge as *B. licheniformis* VSD4 has homologs involved in the *meta*-cleavage pathway of catechol, initiated by catechol 2,3-dioxygenase, while *Pseudomonas* sp. VS38 has homologs for the catechol *ortho*-cleavage pathway, initiated by catechol 1,2-dioxygenase. No enzyme-coding genes (homologs) related to these BTX degradation pathways were found in the genome of *Rhodotorula* sp. VS67.

## 4. Discussion

Due to the chemical coupling of O_3_ and NO*_x_*, the levels of O_3_ and NO_2_ are inextricably linked, and any resultant reduction in the level of NO_2_ is invariably accompanied by an increase in the level of O_3_, and vice versa [49]. HKa,b are both located on the inner ring road of the city of Hasselt, Belgium, with continuous traffic encircled by many tall buildings, HGa,b are located on the outer city ring road with more traffic but away from dense clusters of buildings, and DM and DW are in two nature reserves close-by. So-called street canyon effects could explain site-specific increases in phylloplane BC load, as was observed at HKa,b. In streets with aligned multiple-floor buildings, ventilation and dispersion of local emissions with surrounding and above-rooftop air is hindered [50]. Previously it was shown that the magnitude of street canyon effects depends on street architecture (aspect ratio, heterogeneity, green infrastructure, and openings) [50,51,52], wind conditions, and traffic-induced turbulence [53].

To test if phylloplane BC load influences the bacterial and fungal leaf microbiome of *H. helix*, we assessed the intra- (alpha) and inter-sample (beta) diversity. While fungal alpha diversity was similar under all three phylloplane BC load conditions, alpha diversity was highest for bacterial communities present on leaves with medium and high phylloplane BC loads exposed to greater ambient air pollution. This corroborates with a study on bacterial phylloplane communities of *Platanus* × *acerifolia* trees in Antwerp, Belgium, which showed that alpha diversity was twice as low in the phylloplane sampled in urban green and waterfront locations than adjacent to busy roads, urban residential areas, and industrial sites [54]. Another study conducted on bacterial phylloplane communities associated with seven tree species in Montreal, Canada also concluded that alpha diversity increased with urban intensity [55]. In a study that characterized the phyllospheric bacterial communities of *Carpinus betulus* trees across three locations, (i) the city center of Warsaw, Poland, (ii) a forest in a UNESCO World Heritage Site (Białowieża), (iii) and a forest in one of the world’s oldest operational oil fields (Bóbrka), alpha diversity was again highest in the most urban sampling location, the city center of Warsaw [56]. While these studies were not always able to directly relate increases in urban bacterial diversity to local air pollution parameters, our results indicate that ambient air pollution has the potential to impact bacterial diversity and associated plant/microbe–microbe interactions, plausibly by modifying the phylloplane microhabitat growth conditions and toxicity. This is further demonstrated by our beta diversity analyses, which show that air pollution-induced phylloplane BC load correlates with the diversity of bacterial and fungal *H. helix* leaf communities. Other studies have shown that plants often have microbial leaf communities exhibiting high seasonal variability [57,58,59,60,61], and we show that this is also true in our study over two annual cycles. It is, therefore, remarkable that our results indicate that seasonal effects were not the major driving factor of bacterial and fungal phylloplane diversity. A study on the phylloplane bacterial community of *H. helix* in Antwerp, Belgium also found that leaf communities differed greatly between urban and nonurban locations, where traffic-generated PM was lower [62], and another study that characterized the epiphytic bacterial communities on leaves of *Platanus* × *hispanica* trees suggested that a discriminating environmental effect is related to ultrafine particulate matter (UFP) and BC deposited on leaves [63]. With regard to fungi, one study showed that fungal communities of ornamental plants on roadsides in Sri Lanka adapt to ambient air pollution by shaping a community that is able to degrade aromatic hydrocarbons [64]. Here, we present a strong correlation between phylloplane BC load, increased by ambient air pollution, and bacterial and fungal diversity in a 2 year sampling approach using metabarcoding. We found that the bacterial genera *Novosphingobium*, *Hymenobacter*, and *Methylorubrum*, and the fungal genus *Ampelomyces* were indicators for communities with the highest phylloplane BC load. *Hymenobacter* was shown to dominate the urban phylloplane in two other studies [54,62], and *Ampelomyces quisqualis* was noted, with various intensity, in north-eastern Poland on different species of Erysiphales selected as potential indicators of urban pollution [65].

From a three-stage experiment comprising (i) isolation and (ii) screening of phylloplane microorganisms for diesel fuel metabolization, and (iii) measurements of BTX degradation, we here present two bacteria, *B. licheniformis* VSD4 and *Pseudomonas* sp. VS38, and one yeast, *Rhodotorula* sp. VS67, isolated from an air-polluted environment that are effectively able to degrade benzene, toluene, and/or xylene. Genome analyses revealed genes for the *meta*-cleavage of catechol for *B. licheniformis* VSD4 and for the *ortho*-cleavage of catechol for *Pseudomonas* sp. VS38. Degradation pathways involving catechol *meta*-cleavage are well-documented for several *Bacillus* strains [66,67,68], including *B. licheniformis* [69]. However, only a few *Bacillus* strains are reported as BTX degraders, e.g., *B. subtilis* DM-04 [70] and *B. amyloliquefaciens* W1 [71]. On the other hand, *Pseudomonas* strains are famous for their BTX degradation potential, including *P. putida* F1 [72], *P. putida* YNS1 [73], *P. stutzeri* OX1 [74], and *Pseudomonas* spp. VI4.1 and VI4T1 [75]. Our analyses showed that *Rhodotorula* sp. VS67, a red yeast, does not have the genetic basis to facilitate degradation mechanisms related to the *meta*- and *ortho*-cleavage pathways of catechol. It remains unclear via what mechanism it was able to degrade xylene as the sole carbon source. Since it was able to completely degrade benzene (and almost completely degrade xylene) only when other carbon sources where present, co-metabolization by monooxygenases is most likely involved [76]. The biodegradation of aromatic hydrocarbons by fungi has traditionally been considered to be of a co-metabolic nature [77]. For example, with regard to BTX degradation, the soil fungus *Cladophialophora* sp. T1 was able to degrade toluene and xylene via co-metabolization [78]. Transcriptome analysis of *Cladophialophora immunda* CBS 110551 associated the initial oxidation of the methyl-group of toluene with catalyzation by a membrane-bound cytochrome P450 [79,80].

## 5. Conclusions

Ambient air pollution has wide-ranging and deleterious effects on our environment and is a major issue for the global society. It is evident that air pollution also influences phylloplane microbial communities, as leaf surfaces are continuously exposed to the atmosphere, but the extent and nature of this influence remains unclear. In this field study, over a 2 year period, we sampled *H. helix* leaves at six locations exposed to different ambient air pollution conditions. We showed that ambient air pollution increases the phylloplane black carbon (BC) load 2–7-fold, and that this BC load strongly correlates with the diversity of bacterial and fungal *H. helix* leaf communities, impacting this diversity even more than seasonal effects. We further identified indicators for communities with high, medium, and low phylloplane BC load. Parallel to this, we present one fungal and two bacterial phylloplane strains isolated from an air-polluted environment able to degrade BTX, common airborne pollutants, including a genomics-based description of the degradation pathways involved. The findings of this study indicate that ambient air pollution contributes to shaping bacterial and fungal ivy leaf communities by impacting the diversity and supporting a community which includes members able to degrade airborne pollutants.

## Figures and Tables

**Table 1:** Ambient air pollution parameters. Phylloplane black carbon (BC) load (*n* = 480) and ambient air BC, PM_2.5_, PM_10_, nitrogen dioxide (NO_2_), and ozone (O_3_) concentrations (*n* = 761) are given as mean values ± standard deviation (SD) for the six sampling sites. Low (bcL), medium (bcM), and high (bcH) phylloplane BC load. ^u^ Significantly different (*p* < 0.05) from HGa, ^v^ from HGb, ^w^ from HKa, ^x^ from HKb, ^y^ from DM, and ^z^ from DW. * No. of BC particles (×10^8^) per g of leaf material.

Sampling Site		BC (µg·m^−3^)	PM_2.5_ (µg·m^−3^)	PM_10_ (µg·m^−3^)	NO_2_ (µg·m^−3^)	O_3_ (µg·m^−3^)	Phylloplane BC Load *
HGa	bcM	1.9 ± 1.0 ^vwxyz^	14.1 ± 9.2 ^yz^	22.7 ± 10.4 ^xyz^	34.2 ± 12.5 ^vwxyz^	42.9 ± 21.5 ^vwxyz^	1.9 ± 2.4 ^wxyz^
HGb		1.5 ± 0.8 ^uwxyz^	13.7 ± 9.2 ^yz^	22.2 ± 10.5 ^yz^	28.9 ± 10.6 ^uwxyz^	46.9 ± 22.1 ^uxyz^	1.7 ± 2.3 ^wxyz^
HKa	bcH	1.3 ± 0.7 ^uvyz^	13.0 ± 9.2	21.3 ± 10.4	22.4 ± 9.2 ^uvxyz^	49.9 ± 22.1 ^uz^	4.1 ± 3.8 ^uvyz^
HKb		1.1 ± 0.7 ^uvyz^	12.9 ± 9.2	21.1 ± 10.5 ^u^	20.7 ± 8.9 ^uvwyz^	51.0 ± 22.2 ^uv^	4.4 ± 3.4 ^uvyz^
DM	bcL	0.9 ± 0.6 ^uvwx^	12.1 ± 8.7 ^uv^	19.8 ± 10.0 ^uv^	18.5 ± 7.4 ^uvwxz^	52.1 ± 22.5 ^uv^	0.6 ± 0.7 ^uvwx^
DW		0.9 ± 0.5 ^uvwx^	12.2 ± 8.9 ^uv^	20.1 ± 10.3 ^uv^	16.8 ± 7.7 ^uvwxy^	53.6 ± 22.5 ^uvw^	0.7 ± 0.9 ^uvwx^

## Data Availability

The bacterial and fungal phylloplane metabarcoding samples are available from the Short Read Archive (SRA) of the National Center for Biotechnology Information (NCBI) under project accession number PRJNA706561. Genome assemblies of the phylloplane BTX degraders are available under project accession number PRJNA727619 (*B. licheniformis* VSD4, LMG 32319; *Pseudomonas* sp. VS38, LMG 32320) and PRJNA727621 (*Rhodotorula* sp. VS67, MUCL 58125). Cultures of these isolates are publicly available from the internationally recognized culture collections BCCM/LMG and BCCM/MUCL (accession numbers are underlined).

## Supplementary Materials

The following are available online at https://www.mdpi.com/article/10.3390/microorganisms9102088/s1: Figure S1. Study area and sampling locations; Figure S2. Biennial temperature and precipitation profile; Figure S3. Biennial profile of ambient air pollution parameters; Figure S4. Rarefaction analysis; Figure S5. Seasonal effects on bacterial and fungal phylloplane diversity; Table S1. Genome assemblies of phylloplane BTX degraders.
