# Supplementary material for: Ambient Air Pollution Shapes Bacterial and Fungal Ivy Leaf Communities
Source: Microorganisms. 2021 Oct 3;9(10):2088. doi: 10.3390/microorganisms9102088 (PMC8540654; doi:10.3390/microorganisms9102088)
Supplement: Supplementary file 1 [file microorganisms-09-02088-s001.zip › microorganisms-1404804-supplementary.pdf]

microorganisms

MDPI

Supplementary Materials

Table S1. Genome assemblies of phylloplane BTX degraders. Genome assembly statistics of two bacteria and one yeast isolated from an air-polluted environment that were able to degrade benzene, toluene, and/or xylene (BTX).

	Genome size (Mbp)	Genome cov. (×)	GC cont. (%)	No. of contigs	Contig N50	GenBank assembly accession
B. licheniformis VSD4	4.10	660	46.2	22	1,027,215	GCA_018587905.1
Pseudomonas sp. VS38	6.85	380	61.1	20	1,485,204	GCA_018587945.1
Rhodotorula sp. VS67	20.26	130	60.6	181	350,365	GCA_019059545.1

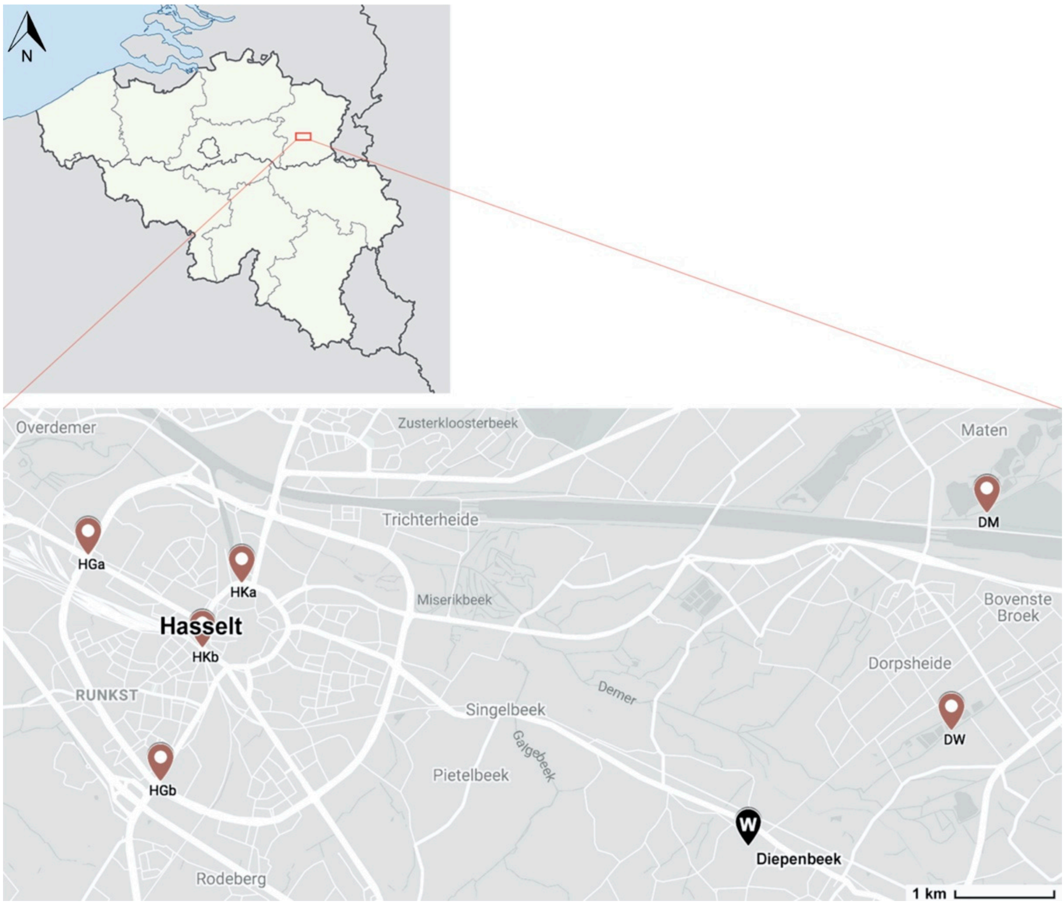

Figure S1. Study area and sampling locations. The six sampling sites (HGa, HGb, HKa, HKb, DM, and DW) around Hasselt, Belgium harboring stable and healthy populations of *H. helix* plants. W: automated weather station owned by the Royal Meteorological Institute of Belgium.

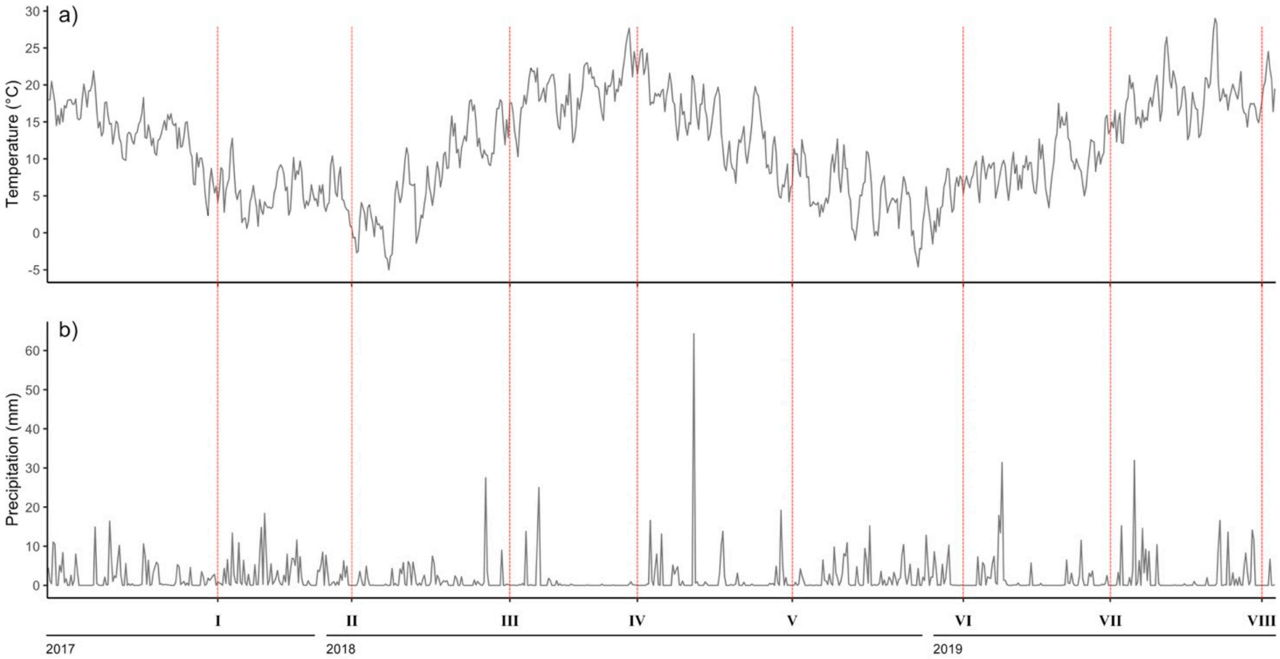

Figure S2. Biennial temperature and precipitation profile. Temperature (**a**) and precipitation (**b**) were monitored daily by an automated weather station from August 1, 2017 until August 31, 2019 (n = 761). Roman numerals indicate the eight sampling events: November 14, 2017 (I); February 5, 2018 (II); May 14, 2018 (III); August 1, 2018 (IV); November 5, 2018 (V); February 19, 2019 (VI); May 21, 2019 (VII); August 23, 2019 (VIII).

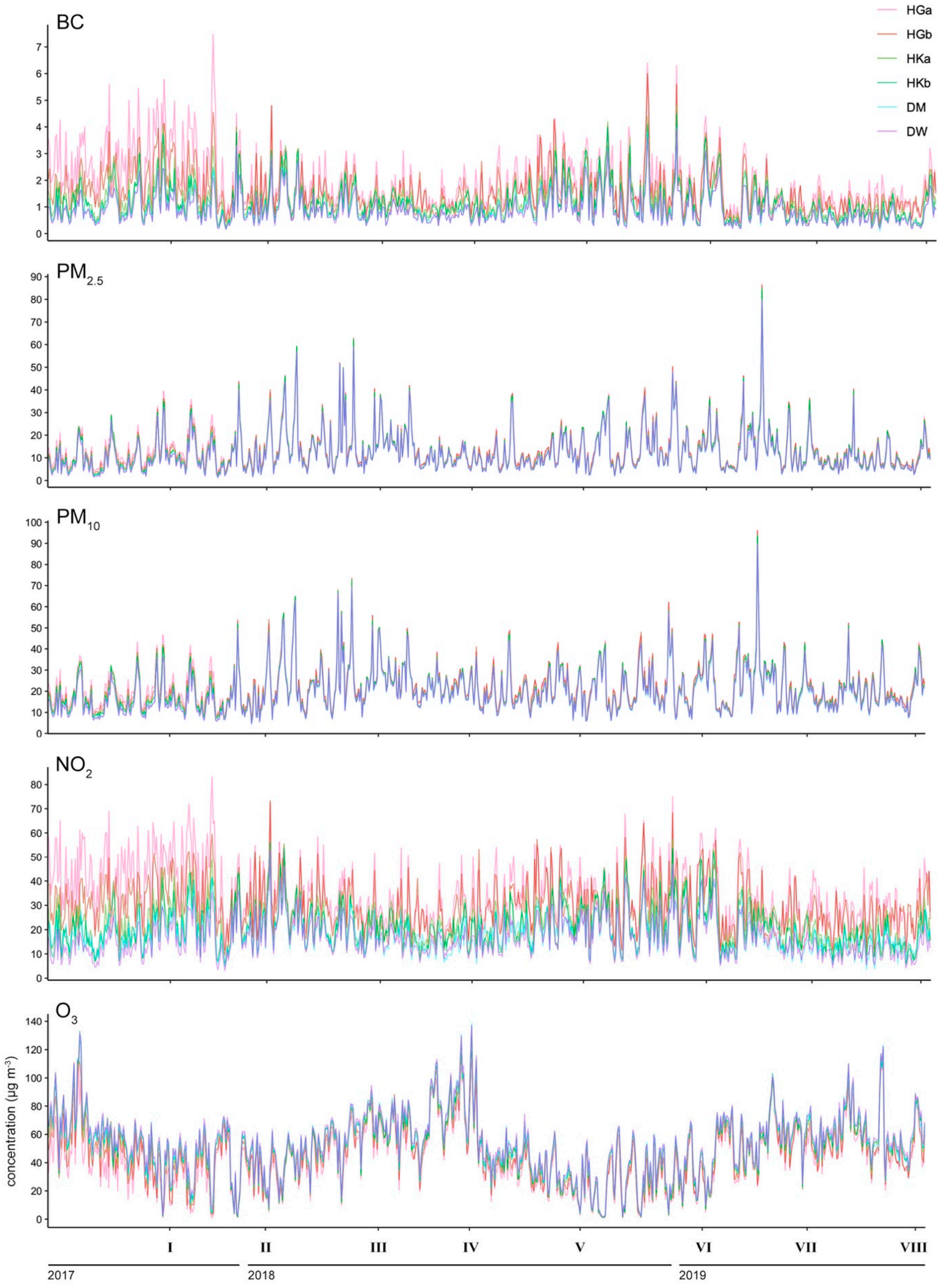

Figure S3. Biennial profile of ambient air pollution parameters. Ambient black carbon (BC), $PM_{2.5}$, PM_{10}, nitrogen dioxide (NO_2), and ozone (O_3) concentrations were monitored daily from August 1, 2017 until August 31, 2019 ($n = 761$) and modelled for every sampling site (HGa, HGb, HKa, HKb, DM, and DW) harboring populations of *H. helix* plants. Roman numerals indicate the eight sampling events: November 14, 2017 (I); February 5, 2018 (II); May 14, 2018 (III); August 1, 2018 (IV); November 5, 2018 (V); February 19, 2019 (VI); May 21, 2019 (VII); August 23, 2019 (VIII).

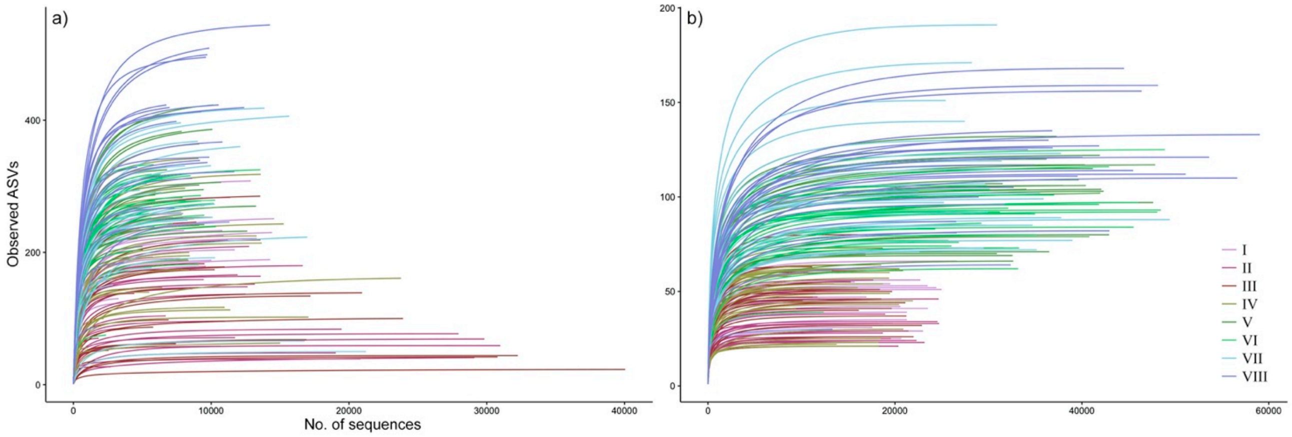

Figure S4. Rarefaction analysis. Rarefaction plots indicating the average number of amplicon sequence variants (ASVs) for bacterial (a; n = 190) and fungal (b; n = 192) samples. Roman numerals indicate the eight sampling events.

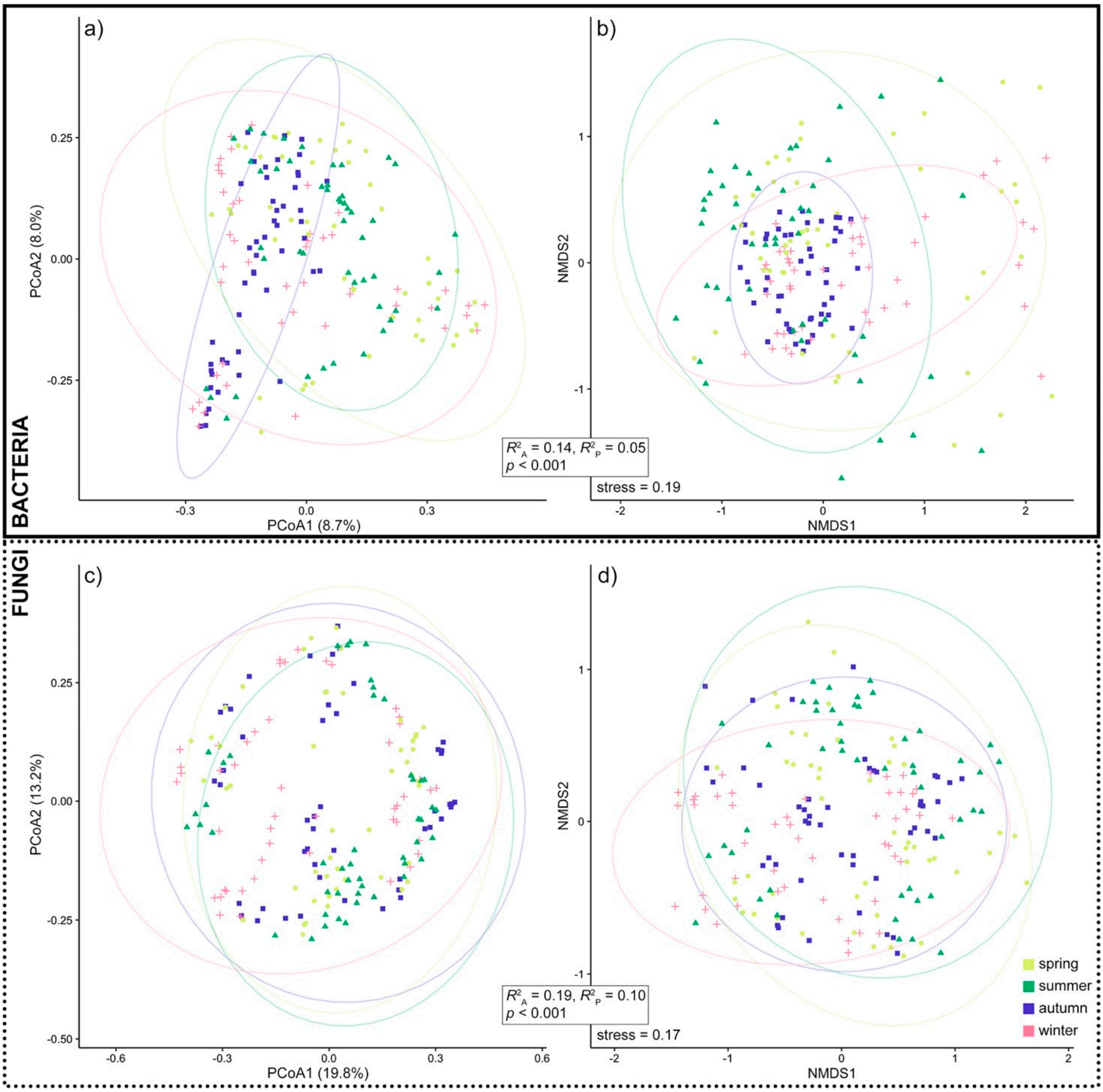

Figure S5. Seasonal effects on bacterial and fungal phylloplane diversity. Bacterial (n = 190) and fungal (n = 192) inter-sample (beta) diversity measured with principal coordinates analysis (PCoA; a,c) and non-metric multidimensional scaling (NMDS; b,d) on Bray–Curtis dissimilarity matrices. Ellipses indicate 95% data intervals. ANOSIM (R^2_A) and PERMANOVA (R^2_P) effect size and significance are shown.
